# Combining ability of highland tropic adapted potato for tuber yield and yield components under drought

**DOI:** 10.1371/journal.pone.0181541

**Published:** 2017-07-25

**Authors:** Betaw Hirut, Hussein Shimelis, Mengistu Fentahun, Merideth Bonierbale, Manuel Gastelo, Asrat Asfaw

**Affiliations:** 1 Sub-Saharan Africa Regional Program, International Potato Centre (CIP), Addis Ababa, Ethiopia; 2 African Centre for Crop Improvement (ACCI), University of KwaZulu-Natal, Pietermaritzburg, KwaZulu-Natal, South Africa; 3 Ethiopian Institute of Agricultural Research (EIAR), Addis Ababa, Ethiopia; 4 Disciplinary Centre of Excellency for Genetics, Genomics and Crop Improvement, International Potato Centre (CIP), Lima, Peru; 5 International Institute of Tropical Agriculture (IITA), Abuja, Nigeria; Louisiana State University College of Agriculture, UNITED STATES

## Abstract

Recurrent drought and late blight disease are the major factors limiting potato productivity in the northwest Ethiopian highlands. Incorporating drought tolerance and late blight resistance in the same genotypes will enable the development of cultivars with high and stable yield potential under erratic rainfall conditions. The objectives of this study were to assess combining ability effects and gene action for tuber yield and traits related to drought tolerance in the International Potato Centre’s (CIP’s) advanced clones from the late blight resistant breeding population B group ‘B3C2’ and to identify promising parents and families for cultivar development. Sixteen advanced clones from the late blight resistant breeding population were crossed in two sets using the North Carolina Design II. The resulting 32 families were evaluated together with five checks and 12 parental clones in a 7 x 7 lattice design with two water regimes and two replications. The experiment was carried out at Adet, in northwest Ethiopia under well-watered and water stressed conditions with terminal drought imposed from the tuber bulking stage. The results showed highly significant differences between families, checks, and parents for growth, physiological, and tuber yield related traits. Traits including marketable tuber yield, marketable tuber number, average tuber weight and groundcover were positively correlated with total tuber yield under both drought stressed and well-watered conditions. Plant height was correlated with yield only under drought stressed condition. GCA was more important than SCA for total tuber yield, marketable tuber yield, average tuber weight, plant height, groundcover, and chlorophyll content under stress. This study identified the parents with best GCA and the combinations with best SCA effects, for both tuber yield and drought tolerance related traits. The new population is shown to be a valuable genetic resource for variety selection and improvement of potato’s adaptation to the drought prone areas in northwest Ethiopia and similar environments.

## Introduction

Recurrent drought is one of the most important constraints to crop growth and productivity in many regions of the world [1, 2]. In Ethiopia, drought is a frequent phenomenon, which threatens food security and rural livelihoods. Agriculture is a major economic sector in the country, employing 85% of the labour force and contributing 48% of the domestic national product [3]. This sector, however, is heavily dependent on the timely onset, amount, duration, and distribution of rainfall [3, 4]. Studies indicate that the area with stable rainfall has decreased, while the area with highly variable rainfall has substantially increased over time [5, 6]. The frequency and severity of this problem is likely to increase as climate change is expected to exacerbate the occurrence of droughts [3, 7, 8]. Lack of irrigation facilities and access to water for agricultural lands across the country make the agricultural system in the country vulnerable to rainfall variability and dry spells. Dry spell probability during the main cropping season (‘*Meher*’) is particularly high at the end of the season [9]. Hence improving drought tolerance in crops is an important strategy to enhance productivity and food security.

Potato (*Solanum tuberosum* L.) has recently become a strategic food security crop in the drought-prone highlands of Ethiopia as its short growth period makes it well suited for crop rotation with other major crops [10, 11]. Moreover, under rain-fed conditions, potato yields more food per unit of water than other major crops [12]. Despite its yield advantages over cereals, potato is more sensitive to water stress than most other crop species due to its sparse and shallow root system [2, 3, 13]. Very little information is available on the actual yield loss that potato experiences due to moisture stress in Ethiopia. However, climate change models have predicted that potato yields will decrease by 15% in Africa by 2030 [3]. Water stress reduces potato growth and production by reducing the amount of productive foliage, by decreasing the rate of photosynthesis per unit of leaf area, and by shortening the vegetative growth period with respect to potato under well-watered conditions [14, 15].

Potato yield under water stress depends on the time, duration, and severity of the stress, as well as on genotype. Decline in photosynthetic rate is fast and substantial, even at relatively high water potentials (-0.3 to -0.5 MPa) [12]. The tuber initiation and bulking stages are those where stress is associated with the highest tuber yield loss [2, 16, 17]. There is genetic variation in the degree to which cultivars are affected by moisture stress, but careful selection strategies are required when breeding for drought tolerance [18]. Results from protected environment studies may not have a direct relevance to drought tolerance in the field. Unexpected shifts in rainfall pattern in drought prone areas, can also reduce the accuracy and effectiveness of phenotyping and phenotypic selection. Phenotyping under field conditions during the dry season may be the best approach to both control the water regime and avoid rainfall disturbance [19, 20].

Breeders seek genotypes that maintain economic yields under water deficit conditions. Important drought tolerance traits must be highly heritable, easy to measure, stable within the measurement period, and without a yield penalty under unstressed conditions [20]. Potato tuber yield is closely related to the ability to intercept solar radiation and to efficiency in dry matter accumulation. Intercepted radiation levels are determined by leaf area [21]. Previous studies [22, 23] showed that groundcover, which is strongly related to leaf area index and biomass, was correlated with tuber yield under both drought and well-watered conditions. Similarly, plant height under water stress can be an important growth parameter to monitor as it has shown a good relationship with drought tolerance [24].

‘Stay green’ or delayed senescence is recognized as important for plant production under terminal drought stress [19, 25]. This trait can be assessed by measuring the chlorophyll content. The Minolta SPAD-502 meter measures green color intensity and is a good indicator of chlorophyll concentration. It is, therefore, an ideal instrument for obtaining data without destructive sampling [26]. In potato, chlorophyll content was reported to have a direct association with drought tolerance, though its contribution to yield was variable [19, 27–29]. Similarly, measuring the canopy temperature has been suggested as a method to identify drought tolerant potato clones as the temperatures are related to stomatal conductance and transpiration, which in turn are associated with the rate of photosynthesis [30].

The ratio of yield reduction by stress, which requires tests under both water stress and non-stress conditions, can also be a useful indicator to select drought tolerant genotypes [19]. Several indices have been suggested to quantify drought tolerance in terms of yield [19, 31, 32]. Stress late in the season favours early maturing genotypes by allowing them to escape the severe, late season stress. The drought tolerance index (DTI) [32] allows a comparison of yield variation under stress that is not confounded by inherent yield potential or early maturity.

Selection of potato genotypes based on combining ability estimates is useful to identify the most valuable parents and families for breeding and cultivar development. The importance of both additive and non-additive gene action in the inheritance of yield and yield components has been reported in different studies under unstressed conditions [33–35]. However, such information is scant under water stressed conditions.

Under Ethiopian fluctuating rainfall condition, drought is often coupled with late blight disease caused by *Phytophthora infestans*, leading to further decline in tuber yield. The disease is widespread in most potato growing areas of the country. Indeed, it has been demonstrated that the disease can causes up to 88% reduction in tuber yield [36]. Selecting individuals and families for drought adaptation in a population improved for late blight resistance could be an important strategy in breeding for yield stability under unpredictable rainfall conditions that cause multiple stresses. The objectives of this study were therefore 1) to determine combining ability effects and gene action governing tuber yield and traits related to drought tolerance in advanced clones from the International Potato Centre’s highland tropics adapted late blight resistant breeding population, and 2) to identify promising parents and crosses for cultivar development.

## Material and methods

### Plant materials

Sixteen potato parental clones from the population B group B3C2 developed by CIP for resistance to late blight with wide genetic background and specific adaptation for the highland tropics [37] were crossed using a North Carolina Design II (NCD II) in two sets [38, 39]. The pedigrees of the parental clones are described in Table 1. Four clones were designated as female and crossed with another four clones used as male parents to form 16 families in each set. In total, 32 families were generated. Twelve of the parental clones and five checks (two advanced clones, namely CIP396038.101 and CIP396029.250 derived from the population B group B3C2 and three widely grown cultivars in Ethiopia, namely Belete, Guassa, and Gorebella) were also included in the study.

**Table 1:** Pedigree of potato parents used to generate families using North Carolina Design II.

Parental clones	Set	Female/Male	Pedigree
CIP395011.2	1	Male	CIP393085.5 x CIP392639.8
CIP396041.102	1	Male	CIP393280.58 x CIP393280.57
CIP395017.229	1	Male	CIP393085.13 x CIP392639.8
CIP396038.107	1	Male	CIP393077.54 x CIP393280.64
CIP395015.6	1	Female	CIP393083.2 x CIP391679.12
CIP395109.34	1	Female	CIP391589.26 x CIP393079.4
CIP396004.263	1	Female	CIP391002.6 x CIP393382.64
CIP396034.103	1	Female	CIP393042.5 x CIP393280.64
CIP395017.14	2	Male	CIP393085.13 x CIP392639.8
CIP395077.12	2	Male	CIP391586.109 x CIP393053.6
CIP396012.288	2	Male	CIP391004.10 x CIP393280.58
CIP396264.14	2	Male	CIP393280.82 x CIP392639.2
CIP395096.2	2	Female	CIP393085.5 x CIP393053.6
CIP395109.7	2	Female	CIP391589.26 x CIP393079.4
CIP395112.32	2	Female	CIP391686.15 x CIP393079.4
CIP396031.108	2	Female	CIP392633.64 x CIP393382.64

### Seedling generation

A maximum of 200 botanical seeds per cross received from the International Potato Centre’s headquarter in Peru was sown in seedling trays filled with a 1:2:1 mixture of sterilised sand, farmyard manure and soil in a screen house at the Adet Agricultural Research Centre (11°17′ N, 37°47′ E and 2240 meters above sea level). After 35 days, 80 to 120 seedlings of each cross were transplanted into one liter plastic pots with dimensions of 13 cm diameter top, 10 cm diameter base, and 10.8 cm depth for further growth. Phosphorus fertilizer in the form of diammonium phosphate was applied at the rate of 690 g m^-3^ and nitrogen at 800 g m^-3^ in the form of urea. Fungicides including Ridomil MZ 72 (8% a.i. metalaxyl + 64% a.i. mancozeb), Bravo (82.5% WP Chlorothalonil), and Mancozeb (80% WP) were sprayed alternatively at two-week intervals. Insecticides including thiamethoxam and diazinon were applied when insects occurred, per the recommendation of the manufacturer.

Tubers from 60 genetically unique seedlings (genotypes) from each of the 32 crosses were harvested separately approximately three months after transplanting. One tuber from each plant was taken to produce tuber families comprised of a single tuber from each genotype. Two sets of tuber families were used for planting in two water regimes, i.e., under well-watered and terminal drought conditions. Seed tubers were stored at Injibara, Ethiopia (10°57′ N, 36°56′ E, 2568 m), in a diffused light storage facility for approximately five months to break tuber dormancy. Subsequently, the first clonal generation of F_1_ families was advanced to a second clonal generation for the next dry season evaluation.

### Trial establishment and experimental design

A total of 49 entries comprising 32 families with 60 progeny each plus their 12 parents along with five checks were planted at the Adet Agricultural Research Centre during the long dry season (November 4, 2014 –March 4, 2015). Trials were established using a 7 x 7 simple lattice design with two replications. All the 32 families from the two sets (without considering the sets) along with 12 parents and 5 checks were randomly allocated to the plots within the design. A plot consisted of three rows of 3 m long, each row having 10 plants. The inter- and intra-row spacing was 0.75 and 0.3m, respectively. Each replication had 30 plants per clone for the parents and checks and 30 different genotypes for the family, totalling 60 clones/genotypes per family. The season was rain free and had a lower mean air temperature than was experienced in the main (rainy) season of 2014. This effect, however, was offset by lower relative humidity and higher wind speed in the dry season that favoured evaporation (Table 2). Two irrigation regimes were applied: well-watered and terminal water stress. Terminal water stress was imposed by withholding water supply six weeks after planting until harvest. The time to impose drought was decided based on previous experience of the time of tuber initiation for most of the parents. Irrigation was applied using furrows. Furrow length was limited to 3.5 m to reduce variation in water infiltration along the furrow.

**Table 2:** Mean, minimum, maximum and average temperatures, relative humidity and wind-run at Adet during the study period in 2014/2015.

	Mean monthly air temperature (°C)				
Months	Maximum	Minimum	Mean	Relative humidity (%)	Wind-run 100 ms^-1^
November	25.7±0.8	9.8±2.0	17.7±1.1	59.0±6.3	0.26±0.08
December	25.7±0.9	7.6±1.6	16.0±0.8	42.0±5.6	0.28±0.08
January	26.9±1.4	6.9±1.7	16.9±1.2	47.5±9.3	0.31±0.06
February	29.9±1.1	8.9±2.2	19.4±1.1	39.3±8.7	0.37±0.08
Mean	27.0±1.97	8.3±2.20	17.5±1.49	46.9±10.37	0.30±0.09

The data are presented as mean ± standard deviation of one month.

**Fig 1:**
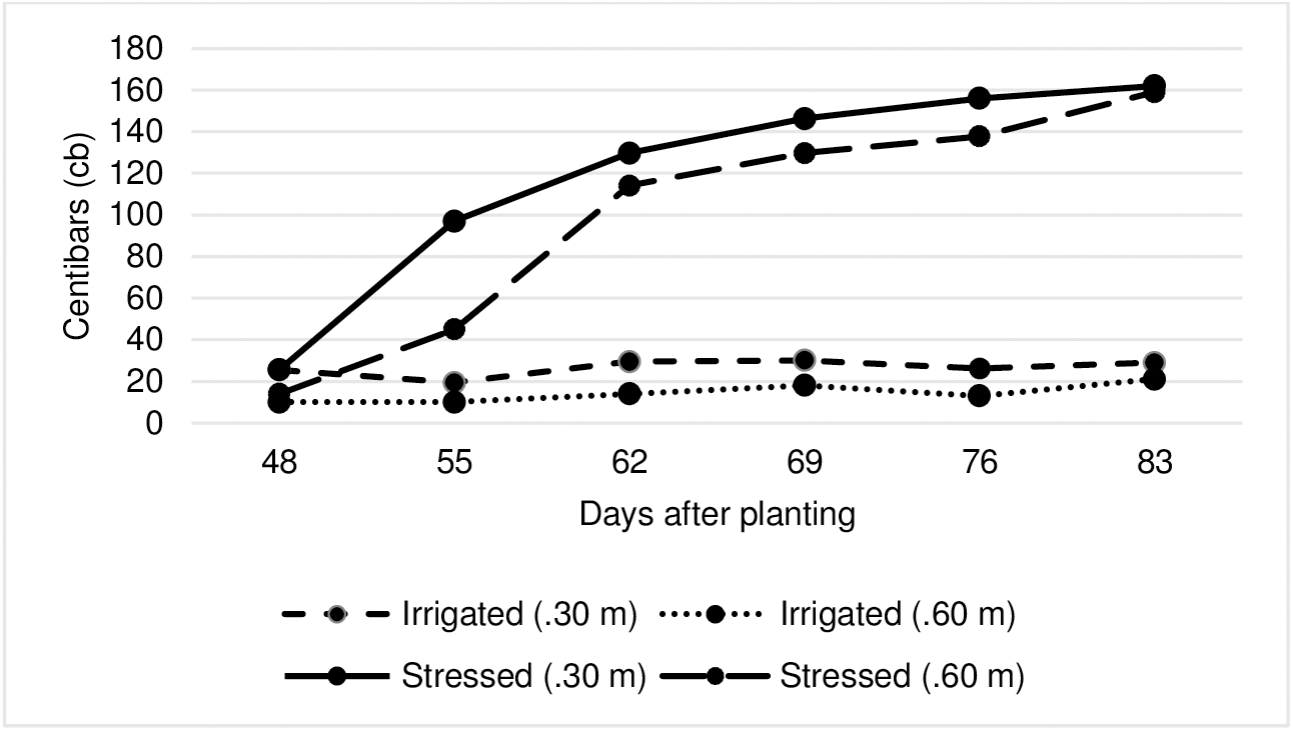
Average soil water potential measured at weekly intervals by water mark sensors at 0.30 m and 0.60 m soil depth on water stressed and non-stressed treatments.

Soil samples were taken randomly from 10 different points of the experimental field at 0 to 30 cm depth in the tilled soil and were mixed thoroughly in a plastic bucket to form one composite sample. The soil analysis was performed at Gondar Soil Laboratory. The soil in Adet was a clay loam with 41% sand, 29% clay, and 30% silt and had electronic conductivity (EC) of 0.18 mS/cm and pH of 6.35. Soil water potential was measured at depths of 0.3 and 0.6 m in both well-watered and water stressed plots with a granular matrix sensor (watermark sensor; Irrometer Co., Box 2424, Riverside, CA 92516, USA). A total of 28 watermark sensors were installed for the experiment: 14 for the well-watered and 14 for the water stressed experiment. Two sensors for every sub-block (row) were installed, one at a depth of 0.3 m and the other at 0.6 m. The well-watered treatment (for the full season) and the water stressed treatment (until the 6^th^ week) were irrigated before the soil water potential at 0.3 m depth reached 30 centibars (cb). According to Shock et al [40], 30 cb is the optimum soil water potential for medium textured soil at 0.3 m depth and 100 to 200 cb indicates that the soil is becoming dangerously dry and production is likely to be adversely affected. The weekly water sensor readings for well-watered and water stressed treatments are presented in Fig 1. Phosphorus fertilizer in the form of diammonium phosphate was applied at the rate of 69 kg ha^−1^ and nitrogen at 81 kg ha^−1^ in the form of urea. The entire dose of phosphorus and half the dose of nitrogen were applied at planting; the other half of nitrogen was added 45 days after planting. Weeding and ridging were carried out by using hoe and hand cultivation. Tubers were harvested 120 days after planting.

### Data collection and analysis

Tuber yield, yield components, growth parameters, and physiological traits were measured under both well-watered and water stressed treatments. For growth and physiological traits, data were collected every two weeks after drought was imposed. Measurements were taken from four tagged plants of parents and checks, and from all individual plants in crosses.

The following growth parameters were measured: plant height (PHT) measured as the length of the main stem between the soil surface and the apex in centimeters; the number of main stems (STN) was counted for each plant; and groundcover percentage (GC) was measured using a grid (0.75 m x 0.60 m) that was divided into (0.075 m x 0.060 m) squares. The quadrat was held just above the canopy and the number of squares at least half filled with green leaves was counted and then divided by the total number of squares to determine the percentage groundcover [21].

Chlorophyll content (CC) was measured using a SPAD-502 chlorophyll meter (Minolta Co., Ltd. Japan) on the two apical leaflets of the third fully expanded leaf of the main stem. Canopy temperature (CT) was measured by a portable infrared thermometer (Major Tech-MT694), which is designed to sense long-wave infrared radiation emitted from its target and convert it to an average temperature that can be related to transpiration. CT measurements (°C) were taken on eight clear (cloudless), windless, and sunny days between 9:00 and 10:00, 10:30 and 11:30, and 12:00 and 13:00 to identify the best time for genotype discrimination.

At harvest, tubers of each plot were graded into two categories: marketable (>30 mm) and unmarketable (rotten, damaged and small tubers <30mm in diameter), which were counted and weighed. From these, the total and marketable number of tubers per plant, total and marketable tuber yield (kg plant^-1^), and average tuber weight (g) were determined. Average tuber weight was calculated as the total tuber yield divided by the total tuber number. The relative reduction of all measured traits was calculated as RR = (control—stressed)/control and expressed as a percentage.

Drought tolerance index (DTI) was determined by multiple regression of stress yield on non-stress yield (yield potential) and number of days from planting to flower bud formation of 50% of plants under well-watered treatments (phenology/earliness) over all genotypes in the study [19, 32] as follows:

$$E(Ys)=a+b_{1}(Yn)+b_{2}(fb)$$

Where: *E*(*Ys*) = the predicted yield under stress; *Yn =* the yield under non stress (yield potential); *fb* = number of days from planting to flower bud formation (phenology) (*fb*); *a* = the intercept; and *b*_1_ and *b*_2_ = the regression coefficients.

The predicted stress yield *E*(*Ys*) for every data point (entry) was subsequently calculated. The actual yield under stress was regressed across all genotypes on the predicted yield under stress. The deviation of the actual (measured) tuber yield under stress from the predicted yield represented the drought tolerance index (DTI). The value of the DTI was then calculated by the studentized residuals [19] from the regression of each data point as depicted below:

$$DTI=\frac{Ys-E(Ys)}{Standard\;error\;of\;E(Ys)}$$

where: *Ys* is the actual tuber yield under stress condition.

High and positive deviations of the actual yield from the predicted yield indicates a relative drought resistance independent of the effect of phenology and yield potential [19].

### Data analysis

The data for growth, physiological and yield related traits were subjected to a general analysis of variance for all crosses, parents, and checks using the GLM procedure of SAS 9.3 [41] statistical program. Two-way analysis of variance was performed in a randomized complete block design (RCBD) because the relative efficiency of the lattice designs over RCBD was not significant. Restricted maximum likelihood estimation of genotypic correlation [42] and Spearman phenotypic correlation were used to examine relationships between traits.

The genetic analysis for the 32 crosses was performed using the North Carolina Design II procedure [39] with SAS version 9.3 [41] using a fixed effects model for individual sets and pooled over sets to identify the significance level of general combining ability (GCA) effects of parents and specific combining ability (SCA) effects of crosses under water stressed conditions. Data were analysed over sets using the following linear model [38]:

$$Y_{ijkp}=µ+S_{p}+g_{i}(S_{p})+g_{j}(S_{p})+h_{ij}(S_{p})+r_{k}(S_{p})+e_{ijkp}$$

Where: *i* = 1, 2, 3; *j* = 1, 2, 3; *k* = 1, 2; *p* = 1, 2; the terms for the model are defined as follows: *Y*_*ijkp*_ denotes the value of a family from the mating between the *i*^th^ female parent, the *j*^th^ male parent, in the *k*^th^ replication within set *p*; μ = grand mean; *S*_*p*_ = the average effect of the *p*^th^ set; *g*_*i*_(*S*_*p*_) = the GCA effect common to all F_1_ families of the *i*^th^ female parent nested within the *p*^th^ set; g_*j*_(*S*_*p*_) = the GCA effect common to all F_1_ families of the *j*^th^ male parent nested within the *p*^th^ set; *h*_*ij*_(*S*_*p*_) = the SCA effect specific to F1 families of the *i*^th^ female and *j*^th^ male parent nested within *p*^th^ set; *r*_*k*_(*S*_*p*_) = the effect of the *k*^th^ replication nested within the *p*^th^ set; and *e*_*ijkp*_ = the random experimental error.

Throughout the text, variation due to males within sets, females within sets, and males x females within sets are referred to as GCA_m_, GCA_f_, and SCA variation, respectively.

For each trait, GCA effect for each clone and SCA effect for each F_1_ family (combination) were calculated according to [43]. For chlorophyll content, negative estimates of GCA, and SCA effects were interpreted as desirable, while neutral or positive estimates were deemed undesirable. For all other traits, positive estimates of GCA and SCA effects were used to identify genotypes with high yield and yield components, whereas neutral or negative estimates were considered undesirable. Standard errors for GCA effects of female and male parents and SCA effects of families in each set were calculated by using the method described by [44].

The relative importance of additive (GCA) and non-additive (SCA) genetic effects in explaining the performance of the progeny for each of the traits was determined by individually expressing the GCAf mean square, GCAm mean square, and the SCA mean square as a percentage of the treatment (crosses) mean square as shown in the formula below [45]:

$$\begin{array}{l} GCA/\left(GCA+SCA\right)\;\%={MS\;GCA}_{pooled}/({MS\;GCA}_{Pooled}+MS\;SCA)\;x\;100\\ {MS\;GCA}_{Pooled}=s(f-1)MS\;{GCA}_{f}+s(m-1)MS\;{GCA}_{m}/s(m+f-2)\;for\;the\;combined\;set\\ {MS\;GCA}_{Pooled}=(f-1)MS\;{GCA}_{f}+(m-1)MS\;{GCA}_{m}/\left(m+f-2\right)\;for\;the\;individual\;sets \end{array}$$

Where; *MS GCA*_*Pooled*_ = mean squares for *GCA*_*Pooled*_; *MS SCA* = mean squares for SCA; *s* = number of sets; *f* = number of female parents; *m* = number of male parents; *MS GCA*_*f*_ = mean square of GCA_f_; *MS GCA*_*m*_ = mean square of GCA_m_, respectively.

## Results

### Genotypic variation under water stressed and well-watered conditions

Highly significant variation (p < 0.001) among entries for all the traits was observed under both stressed and non-stressed conditions (Table 3). For the canopy temperature, significant differences among entries was observed for measurements taken at 10:30 to 11:30 and only this data is reported here. Highly significant interactions between the treatments and entries were observed for most of the traits indicating that entries responded differently according to water level. Significant treatment x entry interactions were observed for most of the growth and physiological traits on the 40^th^ day after drought stress was imposed. Hence the data from this date were used to assess the response of materials (families, parents, and checks) under study. Chlorophyll content showed significant differences among entries and between treatments; however, interaction between entry and treatment was not significant.

**Table 3:** Analysis of variance, mean values, and relative reduction (RR%) of traits under well-watered (control) versus drought conditions.

	Mean square and Significance				Mean trait values		
Traits	Entries (E)^a^	Treatment (T)	E*T	CV (%)	Control	Drought	RR (%)
TTY (kg plant^-1^)	0.031***	5.410***	0.009***	10.9	0.71	0.38	47
MTY (kg plant^-1^)	0.035***	5.816***	0.009***	11.2	0.68	0.34	51
TTN (per plant)	32.832***	47.125***	18.960***	13.2	13.62	14.6	-7
MTN (per plant)	4.633***	176.940***	1.688**	11.9	8.62	6.72	22
ATW (g)	456.550***	36022.670***	165.110***	14.3	55.16	28.05	49
PHT (cm)	230.093***	6108.974***	30.799***	5.6	57.67	46.51	19
GC (%)	246.740***	12159.120***	59.920**	10.3	62.61	46.86	25
STN (per plant)	2.186***	0.004^ns^	0.237^ns^	21.6	2.95	2.96	0
CC (SPAD reading)	37.769***	1623.381***	3.432^ns^	3.5	43.04	48.8	-13
CT(°C)	2.616***	3186.997***	4.419***	5.1	16.9	24.96	-48

TTY, total tuber yield; MTY, marketable tuber yield; TTN, total tuber number; MTN, marketable tuber number; ATW, average tuber weight; PHT, plant height; GC, groundcover; STN, stem number; CC, chlorophyll content; CT, canopy temperature; RR(%), percentage of relative reduction; CV(%), coefficient of variance.

*** *P* < 0.001

** *P* < 0.01

* *P* < 0.05

non-significant at *P* value 0.05.

^a^Entries here refer to sum of families, parents and checks used in this study

### Effects of water stress on potato trait variability

Soil water potential reached 97 cb on the 55^th^ day after planting, ensuring that drought stress coincided with tuber bulking (Fig 1). Drought affected the expression of potato traits assessed under this study, although genotypic responses varied for different traits as observed by trait mean values and the percentage of relative reduction (RR%) (Table 3). Overall, drought stress had a drastic effect on marketable tuber yield, average tuber weight, and total tuber yield with corresponding relative reductions of about 51, 49 and 47%, respectively. The yield reduction in the most susceptible clone, CIP395017.14, was 57% (Table 4). The reductions in groundcover, number of marketable tubers per plant, and plant height due to drought were considerably less, at 25, 22, and 19% reduction, respectively. Canopy temperature increased markedly under water-stress by 48% (Table 3). Chlorophyll content and total tuber number increased slightly by 7 and 13%, respectively, under stress condition. Unsurprisingly, the number of main stems was not affected by drought as stems had emerged before water stress became severe.

**Table 4:** Mean responses of ten traits assessed on families, parents and checks under water stressed condition in 2014/2015 at Adet, Ethiopia.

	TTY^a^	MTY	ATW	PHT	GC	STN	CC	CT	RR	DTI
**Crosses/family**	** **	** **	** **	** **	** **	** **	** **	** **	** **	** **
CIP396034.103 x CIP396038.107	0.46^b^^-^^f^	0.42^b^^-^^g^	27.8^i^^-^^n^	37.0^t^^u^	57.4^a^^-^^d^	3.4^a^^-^^l^	49.2^g^^-^^o^	28.0^p^	14	2.0
CIP395096.2 x CIP396012.288	0.48^a^^-^^e^	0.42^b^^-^^g^	26.5^j^^-^^o^	62.9^a^	58.9^a^^-^^c^	2.7^e^^-^^q^	49.7^h^^-^^q^	23.6^a^^-^^g^	38	1.8
CIP395109.34 x CIP396041.102	0.48^a^^-^^e^	0.45^a^^-^^d^	34.5^f^^-^^i^	56.6^a^^-^^e^	51.9^b^^-^^i^	2.0^m^^-^^q^	52.2^n^^-^^r^	24.3^b^^-^^k^	41	1.7
CIP396031.108 x CIP396012.288	0.41^d^^-^^l^	0.38^c^^-^^j^	35.3^f^^-^^h^	53.4^c^^-^^i^	48.6^d^^-^^m^	3.1^b^^-^^n^	44.3^b^^c^	25.5^f^^-^^o^	35	1.3
CIP395109.34 x CIP396038.107	0.45^b^^-^^g^	0.43^b^^-^^f^	37.0^e^^-^^g^	56.8^a^^-^^e^	49.8^c^^-^^l^	2.2^j^^-^^q^	50.3^j^^-^^q^	24.3^b^^-^^k^	39	1.2
CIP395096.2 x CIP395077.12	0.31^m^^-^^r^	0.27^n^^-^^r^	17.2^r^^-^^t^	43.8^m^^-^^t^	39.2^m^^-^^q^	2.5^g^^-^^q^	52.4^o^^-^^r^	22.9^a^^-^^d^	21	1.1
CIP396031.108 x CIP395017.14	0.38^f^^-^^o^	0.32^i^^-^^p^	20.7^o^^-^^r^	47.7^g^^-^^q^	46.6^e^^-^^n^	3.1^b^^-^^o^	49.1^g^^-^^o^	27.3^o^^p^	40	1.0
CIP395112.32 x CIP396012.288	0.48^a^^-^^e^	0.45^a^^-^^e^	39.7^d^^-^^f^	59.9^a^^-^^c^	59.1^a^^-^^c^	3.2^b^^-^^n^	46.8^c^^-^^i^	24.2^a^^-^^k^	37	0.9
CIP396031.108 x CIP395077.12	0.39^e^^-^^n^	0.36^f^^-^^m^	30.2^g^^-^^k^	45.0^l^^-^^s^	43.4^h^^-^^q^	2.5^g^^-^^q^	49.7^h^^-^^q^	25.3^e^^-^^o^	42	0.8
CIP395112.32 x CIP396264.14	0.44^c^^-^^h^	0.39^c^^-^^i^	23.8^k^^-^^r^	50.5^e^^-^^m^	46.2^f^^-^^n^	4.2^a^^b^	49.1^g^^-^^o^	23.8^a^^-^^h^	45	0.7
CIP395109.7 x CIP395017.14	0.43^c^^-^^j^	0.34^f^^-^^o^	22.1^m^^-^^r^	52.0^d^^-^^l^	54.9^b^^-^^f^	2.6^f^^-^^q^	51.4^l^^-^^r^	23.1^a^^-^^e^	42	0.7
CIP396034.103 x CIP395011.2	0.40^e^^-^^m^	0.33^h^^-^^p^	21.2^n^^-^^r^	43.1^n^^-^^t^	42.1^i^^-^^q^	4.2^a^^b^	49.2^g^^-^^o^	25.9^h^^-^^p^	38	0.4
CIP396004.263 x CIP396038.107	0.31^m^^-^^r^	0.26^o^^-^^r^	18.7^p^^-^^s^	43.4^m^^-^^t^	36.0^o^^-^^q^	2.3^i^^-^^q^	52.1^n^^-^^r^	22.9^a^^-^^d^	38	0.3
CIP395112.32 x CIP395077.12	0.43^c^^-^^i^	0.37^d^^-^^l^	24.9^j^^-^^q^	56.3^a^^-^^f^	51.1^b^^-^^j^	4.1^a^^-^^c^	48.4^f^^-^^l^	24.6^c^^-^^m^	43	0.2
CIP396004.263 x CIP396041.102	0.30^o^^-^^r^	0.27^o^^-^^r^	18.1^q^^-^^s^	43.8^m^^-^^t^	37.5^n^^-^^q^	2.3^i^^-^^q^	53.0^q^^r^	23.3^a^^-^^f^	47	0.1
CIP395109.34 x CIP395017.229	0.38^f^^-^^o^	0.26^p^^-^^r^	12.6^s^^t^	48.8^g^^-^^p^	45.9^f^^-^^o^	2.3^i^^-^^q^	49.7^h^^-^^q^	23.7^a^^-^^h^	51	0.0
CIP396031.108 x CIP396264.14	0.31^n^^-^^r^	0.27^o^^-^^r^	25.2^j^^-^^p^	40.0^r^^-^^t^	39.5^m^^-^^q^	2.2^k^^-^^q^	46.1^c^^-^^g^	25.4^f^^-^^o^	53	-0.1
CIP396004.263 x CIP395011.2	0.34^i^^-^^p^	0.30^j^^-^^q^	24.2^k^^-^^q^	42.9^o^^-^^t^	44.2^g^^-^^p^	4.1^a^^-^^d^	49.3^g^^-^^o^	27.1^n^^-^^p^	48	-0.1
CIP395109.7 x CIP396264.14	0.40^e^^-^^m^	0.36^f^^-^^m^	29.3^h^^-^^l^	52.9^c^^-^^j^	54.9^b^^-^^f^	3.5^a^^-^^j^	48.2^e^^-^^l^	25.3^e^^-^^o^	49	-0.2
CIP396034.103 x CIP396041.102	0.41^d^^-^^k^	0.38^c^^-^^k^	25.2^j^^-^^p^	47.6^h^^-^^q^	53.5^b^^-^^g^	4.6^a^	49.4^g^^-^^p^	25.7^g^^-^^o^	48	-0.2
CIP395109.34 x CIP395011.2	0.30^o^^-^^s^	0.27^o^^-^^r^	27.9^i^^-^^n^	45.4^k^^-^^s^	40.2^k^^-^^q^	2.6^f^^-^^q^	52.9^q^^r^	24.0^a^^-^^i^	44	-0.2
CIP395096.2 x CIP396264.14	0.29^o^^-^^s^	0.27^o^^-^^r^	33.7^f^^-^^i^	46.0^j^^-^^r^	36.7^n^^-^^q^	1.8^o^^-^^q^	52.8^p^^-^^r^	25.9^g^^-^^p^	53	-0.3
CIP395015.6 x CIP395017.229	0.35^h^^-^^o^	0.32^i^^-^^q^	28.3^i^^-^^m^	36.8^t^^u^	43.4^h^^-^^q^	3.4^a^^-^^k^	44.8^b^^-^^e^	25.4^e^^-^^o^	55	-0.4
CIP395109.7 x CIP396012.288	0.41^d^^-^^l^	0.37^e^^-^^l^	36.1^f^^-^^h^	57.2^a^^-^^e^	50.6^b^^-^^j^	4.1^a^^-^^d^	44.4^b^^-^^d^	25.7^g^^-^^o^	52	-0.7
CIP395109.7 x CIP395077.12	0.32^l^^-^^r^	0.29^k^^-^^r^	26.7^j^^-^^o^	46.9^h^^-^^r^	39.4^m^^-^^q^	3.2^b^^-^^m^	47.8^d^^-^^k^	23.9^a^^-^^i^	48	-0.7
CIP395015.6 x CIP396038.107	0.36^h^^-^^o^	0.30^j^^-^^q^	24.3^k^^-^^q^	46.4^i^^-^^r^	46.1^f^^-^^n^	2.9^c^^-^^p^	50.4^k^^-^^q^	26.1^i^^-^^p^	56	-0.8
CIP395112.32 x CIP395017.14	0.35^h^^-^^p^	0.29^l^^-^^r^	18.2^q^^-^^s^	50.2^e^^-^^n^	48.8^d^^-^^m^	3.7^a^^-^^g^	52.3^n^^-^^r^	22.8^a^^-^^c^	55	-1.1
CIP395096.2 x CIP395017.14	0.21^s^	0.17^s^	12.5^s^^t^	46.0^j^^-^^r^	33.5^q^	1.9^n^^-^^q^	54.2^r^	22.8^a^^-^^c^	58	-1.2
CIP396034.103 x CIP395017.229	0.24^r^^s^	0.16^s^	10.5^t^	36.5^t^^u^	35.7^p^^q^	2.6^g^^-^^q^	52.1^n^^-^^r^	26.8^m^^-^^p^	58	-1.2
CIP396004.263 x CIP395017.229	0.25^q^^-^^s^	0.22^r^^s^	20.4^o^^-^^r^	31.2^u^^v^	44.9^f^^-^^p^	2.8^e^^-^^p^	48.6^f^^-^^m^	26.4^k^^-^^p^	64	-1.2
CIP395015.6 x CIP395011.2	0.26^p^^-^^s^	0.21^r^^s^	17.3^r^^-^^t^	46.7^i^^-^^r^	36.0^o^^-^^q^	2.6^f^^-^^q^	49.5^g^^-^^p^	25.1^d^^-^^o^	56	-1.5
CIP395015.6 x CIP396041.102	0.29^o^^-^^s^	0.23^q^^-^^s^	19.3^p^^-^^s^	42.1^p^^-^^t^	38.6^n^^-^^q^	3.1^b^^-^^o^	50.1^i^^-^^q^	26.1^i^^-^^p^	60	-1.5
**Mean of families**	**0.36**	**0.32**	**24.7**	**47.4**	**45.4**	**3**	**49.7**	**24.9**	** **	** **
**Parents and checks**	** **	** **	** **	** **						** **
CIP396038.101*	0.53^a^^b^	0.51^a^	46.7^a^^-^^c^	50.1^e^^-^^o^	49.8^c^^-^^l^	2.7^e^^-^^q^	48.9^g^^-^^n^	23.9^a^^-^^i^	30	2.1
CIP396038.107	0.55^a^	0.53^a^	51.8^a^	55.0^b^^-^^g^	67.3^a^	3.2^b^^-^^n^	46.1^c^^-^^g^	25.3^e^^-^^o^	39	1.9
CIP396029.250*	0.49^a^^-^^d^	0.46^a^^-^^c^	40.0^c^^-^^f^	54.2^b^^-^^h^	41.5^j^^-^^q^	2.4^i^^-^^q^	44.6^b^^-^^d^	26.6^l^^-^^p^	21	1.5
Gorebella*	0.46^a^^-^^f^	0.42^b^^-^^f^	31.7^g^^-^^j^	37.6^t^^u^	52.3^b^^-^^h^	3.8^a^^-^^f^	47.0^c^^-^^j^	25.2^e^^-^^o^	47	0.3
CIP395112.32	0.51^a^^-^^c^	0.48^a^^b^	44.8^b^^d^	60.8^a^^b^	53.0^b^^-^^h^	3.9^a^^-^^e^	51.8^m^^-^^r^	24.7^c^^-^^m^	48	-0.1
Belete*	0.43^c^^-^^j^	0.40^c^^-^^i^	43.1^c^^-^^e^	38.3^s^^-^^u^	50.0^c^^-^^k^	2.8^e^^-^^q^	42.5^a^^b^	27.3^o^^p^	49	-0.1
Guassa*	0.36^h^^-^^o^	0.33^h^^-^^p^	23.7^k^^-^^r^	26.6^v^	38.5^n^^-^^q^	3.6^a^^-^^i^	40.4^a^	26.8^m^^-^^p^	52	-0.1
CIP395017.229	0.39^e^^-^^n^	0.36^f^^-^^m^	28.4^i^^-^^m^	32.5^u^^v^	56.8^b^^-^^d^	2.4^h^^-^^q^	48.1^e^^-^^l^	26.8^m^^-^^p^	53	-0.2
CIP395109.34	0.37^g^^-^^o^	0.36^f^^-^^n^	50.5^a^^b^	58.8^a^^-^^d^	60.5^a^^b^	1.7^p^^q^	52.0^n^^-^^r^	25.5^f^^-^^o^	49	-0.2
CIP396004.263	0.41^d^^-^^k^	0.39^c^^-^^i^	35.8^f^^-^^h^	41.0^q^^-^^t^	44.0^g^^-^^p^	3.7^a^^-^^h^	45.4^b^^-^^f^	24.9^c^^-^^n^	46	-0.5
CIP395096.2	0.30^o^^-^^s^	0.26^p^^-^^r^	19.2^p^^-^^s^	46.8^i^^-^^r^	43.3^h^^-^^q^	1.5^q^	51.2^l^^-^^r^	22.2^a^^b^	50	-0.6
CIP396034.103	0.43^c^^-^^h^	0.41^b^^-^^h^	36.4^e^^-^^g^	49.2^f^^-^^p^	56.3^b^^-^^e^	4.5^a^	48.5^f^^-^^m^	25.5^f^^-^^o^	53	-0.8
CIP396031.108	0.36^g^^-^^o^	0.34^g^^-^^p^	33.8^f^^-^^i^	45.5^k^^-^^s^	49.8^c^^-^^l^	3.1^b^^-^^n^	44.6^b^^-^^d^	24.2^a^^-^^j^	55	-1.0
CIP395015.6	0.34^j^^-^^p^	0.29^l^^-^^r^	27.8^i^^-^^n^	52.6^c^^-^^k^	44.8^g^^-^^p^	2.2^k^^-^^q^	47.0^c^^-^^j^	24.4^b^^-^^l^	48	-1.1
CIP395011.2	0.34^k^^-^^q^	0.28^m^^-^^r^	23.2^l^^-^^r^	31.6^u^^v^	39.5^m^^-^^q^	2.8^d^^-^^p^	46.4^c^^-^^h^	24.2^a^^-^^j^	49	-1.2
CIP395077.12	0.33^k^^-^^q^	0.31^j^^-^^q^	26.2^j^^-^^o^	45.2^l^^-^^s^	40.0^l^^-^^q^	2.4^h^^-^^q^	46.8^c^^-^^i^	26.4^j^^-^^p^	50	-1.3
CIP395017.14	0.33^k^^-^^q^	0.29^l^^-^^r^	22.0^m^^-^^r^	37.6^t^^u^	54.8^b^^-^^f^	2.1^l^^-^^q^	50.4^j^^-^^q^	22.0^a^	57	-1.6
**Mean of parents**	**0.39**	**0.36**	**33.3**	**46.4**	**50.8**	**2.8**	**48.2**	**24.7**		
**Mean of checks**	**0.45**	**0.42**	**37**	**41.3**	**46.4**	**3**	**44.7**	**26**		
**Over all mean**	**0.38**	**0.34**	**28.1**	**46.5**	**46.9**	**3**	**48.8**	**25**		
**CV (%)**	**11.6**	**12.3**	**12.2**	**7.8**	**10.6**	**21.3**	**3.5**	**4.5**	** **	** **

TTY, total tuber yield (kg plant^-1^); MTY, marketable tuber yield (kg plant^-1^); ATW, average tuber weight (g); PHT, plant height (cm); GC, groundcover (%); STN, stem number (per plant); CC, chlorophyll content (SPAD reading); CT, canopy temperature (°C); RR, relative reduction of total tuber yield due to drought stress (%); DTI, drought tolerance index.

^a^^-^^z^ Means in a column followed by the same letter(s) are not significantly different at *p* ≥ 0.05.

*clones used as checks, the rest are parents.

### Genotypic correlations of tuber yield, growth and physiological traits under drought and well-watered conditions

Table 5 presents genotypic correlation of tuber yield and growth traits measured under drought and well-watered conditions. Total tuber yield from both drought and well-watered treatments had significant and positive correlation with marketable tuber yield, marketable tuber number, average tuber weight, and groundcover. Plant height was positively correlated (*r* = 0.65) with yield only under water stressed condition. On the other hand, the number of stems per plant showed a strong and positive correlation (*r* = 0.74) with yield only under well-watered conditions.

**Table 5:** Genotypic correlation between yield, growth and physiological parameters on tuber families, parental clones and checks grown under water stressed (below diagonal) and well-watered (above diagonal) conditions at Adet, Ethiopia in 2014/15.

Triats	CT	CC	GC	PHT	STN	ATW	MTN	TTN	MTY	TTY
CT		0.50±0.13**	-0.34±0.17*	0.30±0.15*	-0.55±0.16**	-0.19±0.17	-0.73±0.13**	-0.13±0.18	-0.52±0.14**	-0.52±0.14**
CC	-0.52±0.15**		-0.30±0.16	0.24±0.14	-0.51±0.15**	-0.23±0.15	-0.45±0.14**	0.01±0.17	-0.40±0.14**	-0.38±0.14*
GC	0.14±0.20	-0.15±0.18		0.35±0.15*	0.58±0.15**	0.37±0.16*	0.68±0.12**	0.14±0.18	0.88±0.06**	0.91±0.06**
PHT	-0.33±0.17*	0.22±0.16	0.43±0.14**		-0.34±0.17*	0.22±0.15	-0.15±0.16	-0.08±0.16	0.23±0.15	0.25±0.15
STN	-0.70±2.99	-0.50±2.21	1.763±6.97	1.595±5.87		0.07±0.20	0.92±0.12**	0.40±0.18*	0.72±0.14**	0.74±0.14**
ATW	0.30±0.17	-0.39±0.14**	0.62±0.12**	0.38±0.14**	-0.08±0.84		-0.05±0.18	-0.71±0.09**	0.66±0.10**	0.61±0.11**
MTN	0.19±0.25	-0.50±0.21*	0.55±0.18**	0.36±0.19	1.780±6.03	0.27±0.21		0.43±0.15**	0.65±0.12**	0.68±0.11**
TTN	-0.10±0.20	0.28±0.17	-0.38±0.17*	-0.31±0.16	0.73±3.38	-0.80±0.08**	0.02±0.23		-0.07±0.17	0.02±0.17
MTY	0.22±0.19	-0.40±0.15**	0.79±0.08**	0.48±0.13**	1.055±4.11	0.83±0.06**	0.70±0.13**	-0.50±0.14**		0.99±0.01**
TTY	0.08±0.22	-0.36±0.19	0.86±0.10**	0.65±0.12**	1.057±3.53	0.68±0.13**	0.80±0.09**	-0.24±0.19	0.98±0.03**	

CT, canopy temperature (°C); CC, chlorophyll content (SPAD reading); GC, groundcover (%); PHT, plant height (cm); STN, stem number (per plant); ATW, average tuber weight (g); MTN, marketable tuber number (per plant); TTN, total tuber number (per plant); MTY, marketable tuber yield (kg plant^-1^); TTY, total tuber yield (kg plant^-1^).

*, ** significantly different from zero at ≥ 1.96 and 2.56 standard error, respectively

Canopy temperature did not show a strong correlation with most of the traits measured under water stressed condition (Table 5). However, under well-watered conditions, canopy temperatures showed negative and highly significant genotypic correlations with the number of main stems per plant, number of marketable tubers, marketable and total tuber yields (*r* = -0.55, -0.73, -0.52 and -0.52, respectively).

Strong negative correlations were obtained between chlorophyll content and most yield components, i.e., average tuber weight, marketable tuber number, and yield under drought. Under well-watered treatment, chlorophyll content had a significant and negative correlation with the number of stems per plant, marketable tuber number, marketable and total tuber yield.

### Trait values and level of drought tolerance of parental clones, tuber families and checks

Data for tuber yield and seven important yield determinant traits that showed correlation to total tuber yield are presented in Table 4. Families, parents, and checks in Table 4 are sorted in descending order based on their drought tolerance index (DTI) values. The phenotypic correlation between drought tolerance index (DTI) and yield under water stressed condition was positive (*r* = 0.60), whereas the index showed no correlation (*r* = -0.184) with yield under well-watered condition. The total tuber yield per plant for families ranged from 0.5 (CIP395109.34 x CIP396041.102) to 0.2 kg (CIP395096.2 x CIP395017.14), while for the parents and checks, it ranged from 0.6 (CIP396038.107) to 0.3 kg (CIP395096.2). The clones CIP396038.101, CIP396038.107, CIP396029.250, Gorebella, and CIP395112.32 were the most tolerant based on drought tolerance index. All of these clones have relatively high plant height and groundcover except Gorebella (short plant height) and CIP396029.250 (lower groundcover value). Clone CIP396038.107 had the highest groundcover and favorable chlorophyll content (lower). Clone CIP395112.32 showed the highest stem number and plant height. The clones with high DTI differed widely in their canopy temperatures. The following families displayed high yield under water stress and high drought tolerance index: CIP396034.103 x CIP396038.107, CIP395096.2 x CIP396012.288, CIP395109.34 x CIP396041.102, CIP396031.108 x CIP396012.288, CIP395109.34 x CIP396038.107, CIP395112.32 x CIP396012.288, CIP395112.32 x CIP396264.14, CIP395109.7 x CIP395017.14, CIP396034.103 x CIP395011.2, and CIP395112.32 x CIP395077.12.

### Combining ability

The genetic analyses of variances of groundcover, plant height, marketable and total tuber yield, average tuber weight, and chlorophyll content under water stress, following a North Carolina Design II procedure pooled over sets, is presented in Table 6. Homogeneity of error variances in the two sets was observed from Bartlett’s test [46] for the traits considered in the study. Results showed that the GCA due to females within sets (GCA_f_), GCA due to males within sets (GCA_m_) and SCA within sets were significant for all traits tested. The sets were significantly different for all traits.

Total GCA (i.e., male plus female main effects) accounted for 83% of plant height and chlorophyll content, 68% of marketable and average tuber weight, 66% of total tuber yield, and 60% of groundcover. Hence, GCA were more important than SCA variances for all traits. Contribution of GCA_f_ and GCA_m_ were similar for most of the traits assessed. However, the GCA_m_ for average tuber weight, marketable tuber weight, and chlorophyll content were 3, 1.6 and 1.5 times larger than GCA_f_ variances, respectively.

**Table 6:** Summary of mean squares and significant tests of combining ability effects for tuber yield and growth traits under water stressed conditions for potato families evaluated at Adet, Ethiopia.

Source of variation	d.f	TTY	MTY	ATW	GC	PHT	CC
Set	1	0.0125*	0.018**	187.80***	144.43*	695.40***	6.13*
Replication (set)	2	0.0003^ns^	0.002^ns^	104.18***	64.95^ns^	173.14***	13.10**
GCA_f_	6	0.0160***	0.014***	77.56***	128.44***	158.50***	20.32***
GCA_m_	6	0.0147***	0.022***	235.51***	135.45***	171.05***	30.89***
SCA	18	0.0079***	0.008***	74.39***	88.16***	32.63***	5.32***
Error	30	0.0017	0.001	3.06	23.95	8.29	0.92
GCA/(GCA+SCA)%		65.992	68.39	67.79	59.95	83.47	82.81
Contribution of GCAf		34.340	26.43	16.79	29.18	40.15	32.86
Contribution of GCAm		31.652	41.95	50.99	30.77	43.32	49.95

d.f., degrees of freedom; TTY, total tuber yield (kg plant^-1^); MTY, marketable tuber yield (kg plant^-1^); ATW, average tuber weight (g); GC, groundcover (%); PHT, plant height (cm); CC, chlorophyll content (SPAD reading); GCA, general combining ability; GCA_f_, general combining ability for female, GCA_m_, general combining ability for male; SCA, specific combining ability.

*** *p* < 0.001

** *p* < 0.01

* *p* < 0.05

non-significant at *p* value 0.05.

Analysis of individual sets showed that GCA_m_ was significantly larger than GCA_f_ variance only for chlorophyll content in set I (Table 7). Generally, the proportions of GCA variance over total treatment variance were higher in set II than in set I for all traits. Set II had larger mean values for total tuber yield, marketable tuber yield, average tuber weight, groundcover, plant height, and low mean value for chlorophyll content. This indicates that more drought tolerant individuals could be found in set II than in set I. GCA_f_ variance was relatively larger than GCA_m_ variance for most of the traits evaluated in set I, whereas GCA_m_ was relatively larger than GCA_f_ in set II for all traits except chlorophyll content. This reflects female parents in set I and male parents in set II differing more in the traits measured than their male and female counterparts, respectively. GCA_f_ was non-significant for chlorophyll content in set I. SCA was non-significant for chlorophyll content in set II.

**Table 7:** Summary mean squares and significance tests of combining ability effects for yield and growth traits under water stress conditions on potato families tested in set I and set II at Adet, Ethiopia.

Source of variation	d.f.	TTY	MTY	ATW	GC	PHT	CC
Set I							
Replication	1	0.0042^ns^	0.0070^ns^	86.81***	1.334^ns^	100.77*	5.23^ns^
GCA_f_	3	0.0186***	0.0154***	95.86***	103.679^ns^	228.16***	0.71^ns^
GCA_m_	3	0.0135**	0.0218***	114.11***	70.696^ns^	129.11***	23.15***
SCA	9	0.0084**	0.0110***	102.39***	88.735^ns^	36.26*	8.15**
Error	15	0.0017	0.0017	2.15	35.778	12.13	1.53
CV (%)		11.778	13.5466	6.39	13.612	7.9	2.81
Mean		0.3495	0.3006	22.95	43.941	44.07	44.01
R^2^		0.8739	0.8972	0.98	0.711	0.89	0.87
GCA/(GCA+SCA)%		65.5515	62.8355	50.63	49.56	83.12	59.4
GCAf/GCAm ratio		1.3768	0.7059	0.84	1.467	1.77	0.03**
Set II							
Replication	1	0.0018^ns^	0.0006^ns^	26.19*	104.904**	73.44**	8.02*
GCA_f_	3	0.0133**	0.0118***	59.27***	153.191***	88.84***	39.93***
GCA_m_	3	0.0159***	0.0214***	356.91***	200.199***	212.98***	38.62***
SCA	9	0.0074**	0.0053**	46.39***	87.584***	29.00**	2.48^ns^
Error	15	0.0015	0.001	3.6	10.958	4.92	1.03
CV (%)		10.292	9.646	7.19	7.051	4.38	2.34
Mean		0.3775	0.3339	26.38	46.946	50.66	43.39
R^2^		0.8732	0.9047	0.97	0.922	0.94	0.95
GCA/(GCA+SCA)%		66.4819	75.8768	81.77	66.859	83.88	94.06
GCAf/GCAm ratio		0.8377	0.553	0.17	0.765	0.42	1.03

d.f., degree of freedom; TTY, total tuber yield (kg plant^-1^); MTY, marketable tuber yield (kg plant^-1^); ATW, average tuber weight (g); GC, groundcover (%); PHT, plant height (cm); CC, chlorophyll content (SPAD reading); GCA, general combining ability; GCA_f_, general combining ability for female; GCA_m_, general combining ability for male; SCA, specific combining ability; R^2^, coefficient of determination.

significant at

*** *p* < 0.001

** *p* < 0.01

* *p* < 0.05

non-significant at *p* value 0.05.

### General combining ability effects of parents

Estimates of the GCA effects for the 16 parents are shown in Table 8. The GCA estimates for total tuber yield ranged from 0.052 for clone CIP395109.34 to -0.048 for clone CIP396004.263 in set I and from 0.065 for clone CIP396012.288 to -0.052 for clone CIP395096.2 in set II. The parents that possessed good GCA effects for tuber yield under drought stress were CIP395109.34, CIP396034.103 and CIP395112.32 from the females and CIP396012.288 and CIP396038.107 from the males; all had significant and positive estimates. These parents also showed positive GCA estimates for marketable tuber yield, average tuber weight, groundcover, and plant height under drought conditions. Male parent CIP396012.288 had the highest positive GCA effect for yield and agronomic traits and female parent CIP396034.103 showed a negative GCA effect for chlorophyll content. Parent CIP396038.107 had positive (undesirable) GCA for chlorophyll content. Parents CIP396004.263, CIP395096.2 and CIP395017.14 displayed undesirable GCA for all traits measured. Parents CIP395015.6 and CIP395017.229 also had undesirable GCA for most of the traits although the latter had significant desirable effect for chlorophyll content.

**Table 8:** Estimates of general combining ability (GCA) effects of 32 potato parents in two sets for six traits assessed at Adet, Ethiopia.

Parents	TTY	MTY	ATW	GC	PHT	CC
Set I						
Female						
CIP395015.6	-0.034**	-0.035**	-0.657	-2.932	-1.056	-0.314
CIP395109.34	0.052**	0.052**	5.053**	3.019	7.827**	0.247
CIP396004.263	-0.048**	-0.038**	-2.601**	-3.297	-3.747**	0.261
CIP396034.103	0.029*	0.02	-1.795**	3.21	-3.024**	-0.195
Male						
CIP395011.2	-0.024	-0.021	-0.29	-3.319	0.461	0.286
CIP395017.229	-0.044**	-0.063**	-5.015**	-1.465	-5.740**	-2.413**
CIP396038.107	0.045**	0.052**	3.974**	3.368	1.829	1.570**
CIP396041.102	0.023	0.032**	1.330**	1.415	3.451**	0.557
SE	0.013	0.012	0.449	1.831	1.066	0.379
Set II						
Female						
CIP395096.2	-0.052**	-0.050**	-3.914**	-4.884**	-1.008	3.041**
CIP395112.32	0.046**	0.043**	0.282	4.323**	3.550**	0.314
CIP395109.7	0.012	0.008	2.154**	2.995**	1.598*	-1.656**
CIP396031.108	-0.005	-0.001	1.478*	-2.435*	-4.140**	-1.699**
Male						
CIP395017.14	-0.036**	-0.054**	-8.020**	-1.013	-1.701*	2.150**
CIP395077.12	-0.012	-0.009	-1.629**	-3.670**	-2.657**	-0.146
CIP396012.288	0.065**	0.070**	8.012**	7.322**	7.675**	-2.981**
CIP396264.14	-0.017	-0.008	1.637**	-2.639**	-3.317**	0.977**
SE	0.012	0.01	0.581	1.014	0.679	0.31

TTY, total tuber yield (kg plant^-1^); MTY, marketable tuber yield (kg plant^-1^); ATW, average tuber weight (g); GC, groundcover (%); PHT, plant height (cm); CC, chlorophyll content (SPAD reading); SE, standard error.

*, ** significantly different from zero at ≥ 1.96SE and 2.56SE, respectively.

### Specific combining ability effects and mean response of families

Families from the following crosses showed significant and desirable SCA effects for most of the traits measured: CIP395109.34 x CIP396041.102, CIP395015.6 x CIP395017.229, and CIP396004.263 x CIP395011.2 in set I, and CIP395096.2 x CIP396012.288, CIP395109.7 x CIP395017.14, and CIP396031.108 x CIP395017.14 in set II (Table 9). The cross CIP396034.103 x CIP395011.2 also had a significant and desirable SCA for total tuber yield. Among these families, CIP395109.34 x CIP396041.102, CIP395096.2 x CIP396012.288, CIP395109.7 x CIP395017.14, and CIP396031.108 x CIP395017.14 were drought tolerant with the highest DTI value.

**Table 9:** Estimates of specific combining ability (SCA) of 32 F1 potato families evaluated in two sets for yield, yield components and drought related traits at Adet, Ethiopia.

Crosses	TTY	MTY	ATW	GC	PHT	CC
Set I						
CIP395015.6 x CIP395011.2	-0.029	-0.036	-4.722**	-1.69	3.255*	0.095
CIP395015.6 x CIP395017.229	0.082**	0.114**	11.028**	3.844	-0.464**	-2.752**
CIP395015.6 x CIP396038.107	-0.003	-0.014	-1.932**	1.722	1.549	1.13
CIP395015.6 x CIP396041.102	-0.049**	-0.063**	-4.374**	-3.875	-4.341**	1.528**
CIP395109.34 x CIP395011.2	-0.080**	-0.058**	0.211	-3.407	-6.929**	0.617
CIP395109.34 x CIP395017.229	0.02	-0.032	-10.388**	0.428	2.609	-0.198
CIP395109.34 x CIP396038.107	0.003	0.02	4.978**	-0.551	3.077	-0.142
CIP395109.34 x CIP396041.102	0.057**	0.069**	5.200**	3.53	1.243	-0.276
CIP396004.263 x CIP395011.2	0.063**	0.062**	4.150**	6.83	2.124	0.634
CIP396004.263 x CIP395017.229	-0.008	0.019	5.055**	5.748	-3.348**	-1.137*
CIP396004.263 x CIP396038.107	-0.034	-0.051**	-5.668**	-8.018	1.244	-0.146
CIP396004.263 x CIP396041.102	-0.021	-0.029	-3.537**	-4.56	-0.02	0.65
CIP396034.103 x CIP395011.2	0.046*	0.032	0.361	-1.732	1.55	-1.345*
CIP396034.103 x CIP395017.229	-0.093**	-0.101**	-5.695**	-10.02	1.202	4.088**
CIP396034.103 x CIP396038.107	0.035	0.045*	2.623**	6.847	-5.871**	-0.841
CIP396034.103 x CIP396041.102	0.013	0.023	2.711**	4.905	3.118	-1.901**
**SE**	0.019	0.019	0.686	2.798	1.629	0.579
Set II						
CIP395096.2 x CIP395017.14	-0.076**	-0.062**	-1.961*	-7.567**	-1.987	0.671
CIP395096.2 x CIP395077.12	0.001	0	-3.626**	0.822	-3.232**	-0.643
CIP395096.2 x CIP396012.288	0.089**	0.065**	-4.007**	9.505**	5.589**	-0.579
CIP395096.2 x CIP396264.14	-0.014	-0.003	9.594**	-2.76	-0.37	0.551
CIP395112.32 x CIP395017.14	-0.037*	-0.034*	-0.46	-1.465	-2.305*	1.298
CIP395112.32 x CIP395077.12	0.018	0.004	-0.087	3.456*	4.754**	-0.174
CIP395112.32 x CIP396012.288	-0.011	0.005	5.030**	0.464	-2.023	0.254
CIP395112.32 x CIP396264.14	0.03	0.025	-4.483**	-2.455	-0.426	-1.378
CIP395109.7 x CIP395017.14	0.072**	0.056**	1.534	5.981**	1.409	-0.05
CIP395109.7 x CIP395077.12	-0.053**	-0.042**	-0.225	-6.882**	-2.655*	0.536
CIP395109.7 x CIP396012.288	-0.047**	-0.044**	-0.449	-6.686**	-2.721**	-0.687
CIP395109.7 x CIP396264.14	0.028	0.029	-0.86	7.587**	3.968**	0.202
CIP396031.108 x CIP395017.14	0.040*	0.039**	0.887	3.051*	2.883**	-1.919
CIP396031.108 x CIP395077.12	0.034	0.038*	3.938**	2.603	1.134	0.281
CIP396031.108 x CIP396012.288	-0.03	-0.026	-0.573	-3.283*	-0.845	1.013
CIP396031.108 x CIP396264.14	-0.044*	-0.051**	-4.251**	-2.372	-3.172**	0.625
**SE**	0.018	0.015	0.887	1.548	1.038	0.474

TTY, total tuber yield (kg plant^-1^); MTY, marketable tuber yield (kg plant^-1^); ATW, average tuber weight (g); GC, groundcover (%); PHT, plant height (cm); CC, chlorophyll content (SPAD reading); SE, standard error.

*, ** significantly different from zero at ≥ 1.96SE and 2.56SE, respectively.

## Discussion

### Drought effects

Drought reduced the tuber yield of all potato families, parental clones, and checks in the present study. The potatoes had reduced marketable tuber number and yield, tuber size, plant height, and groundcover due to drought. Contrary to previous reports [22, 47, 48], the total tuber number increased by 7% under drought stress. It has previously been reported that in both controlled and field conditions, drought before tuber initiation increased total tuber number, while the number of tubers remained unchanged when drought occurred during tuber initiation [49]. Yield under drought stress did not show any relation with the total tuber number in the present study, indicating that yield reduction due to the stress was associated instead, with reduction of tuber size. Drought caused an undesirable reduction in tuber size, which decreased the number of marketable tubers (>30mm in diameter). This was confirmed by the relatively strong and negative correlation of total tuber number with average tuber weight (*r* = -0.80) under water stress as compared to the same correlation under well-watered condition (*r* = -0.71). A similar result was reported earlier [50]. It has also been reported that total tuber number was a poor predictor of tuber yield under stress [47].

Chlorophyll content, which can be used as an indicator of delayed senescence, increased significantly (13%) in response to water stress. Similar results have previously been reported in potatoes [25, 27]. The increase in chlorophyll content might be explained by turgor loss or by a reduction of leaf growth [25, 51]. Reduction of leaf growth was confirmed by a 25% reduction in groundcover under stress (Table 2). However, the entries (families, parental clones, and checks) x water level interaction was non-significant, showing that genotypes had increased their chlorophyll content under stress in a similar manner. Previous reports pointed out that ‘stay green’ (non-senescence) is largely a constitutive trait that can be expressed under well-watered conditions, and is highly heritable, which makes it an easily manipulated trait for breeding [19, 25]. Canopy temperature increased due to stress. Decreased water uptake due to soil water depletion closes stomata, which reduces transpiration and increases leaf temperature [52]. Leaf-canopy temperature is a reliable indicator of plant water stress [19, 53].

### Relation between yield and other traits assessed

High yielding families, parental clones, and checks under water stress condition were better able to maintain their marketable tuber yield, marketable tuber number, tuber size, plant height, and groundcover. Among the secondary traits tested, groundcover seems to be the major determinant of yield under stress followed by plant height (r = 0.86 and 0.65, respectively). A similar result has been reported using vegetative indices, which are related to leaf area index and above ground biomass [22]. Prior studies have also shown that genotypes that exhibit less reduction in growth and carbon assimilation rate under stress exhibit less tuber yield reduction [21, 25]. The measurement of groundcover has the advantage of being quick and non-destructive.

In the present study, loss of leaf chlorophyll had a significant relationship with high tuber size, marketable tuber number, and yield under stress. Loss of chlorophyll in pea and soybean was related to resource remobilization towards seed filling under drought [19]. In a previous study [27], a slower rate of chlorophyll reduction and increased leaf greenness at early senescence were negatively correlated with tuber yield under water restriction in potato. This suggests that the capacity for resource mobilization to harvested plant organs, which leads to higher yield, is mutually exclusive with stay-green [19]. Contrasting results, where maintenance of chlorophyll content is associated with improved yield, have also been reported previously [54].

The high yielding clones tended to be cooler under the well-watered condition, although no association was observed between canopy temperature and yield under water stress. Low canopy temperature is related to large stomatal conductance and transpiration, which is associated with an increased rate of photosynthesis. Early stomatal closure in response to drought causes a reduction in growth rate and final yield [25, 30]. In a previous study, higher yielding wheat genotypes under different soil moisture condition showed lower canopy temperature under well-watered environment [55]. Selection for low-transpiration types may translate to selection for low yield depression under stress but would result in lower yields under optimum conditions [15]. The absence of correlation between canopy temperatures versus yield under water stress suggests that yield improvement in a water-limited environment is not necessarily associated with traits that cause a yield penalty in a high yielding environment.

### Variation in drought tolerance among the genotypes tested

Among several phenological measurements taken in well-watered treatments in the present study (S3 Table), the number of days from planting to 50% of plants exhibiting flower bud formation showed significant (*r* = -0.57) correlation with tuber yield, revealing that early flowering genotypes had a yield advantage over the late flowering ones. A highly significant (*r* = 0.60) correlation was also observed between yield under the well-watered condition and yield under stress. Given the strong effects of phenology and yield potential on yield under the stress in this study, it is clear that tuber yield under stress *per se* is of little value in describing a genotype’s drought tolerance. Regression analysis of the combined effects of phenology and yield potential on tuber yields in the stress treatments indicated that these two factors accounted for 45.5% of the observed variation in tuber yield under stress (S4 Table). Therefore, in this study a drought tolerance index (DTI), which corrects the differential tuber yield performance under stress for variation to number of days from planting to flower bud formation and unstressed yield, was used to identify drought tolerant genotypes and crosses. This index can help assure that the yield achieved under stress was due to drought tolerance, instead of drought escape and is independent of yield potential.

The DTI was positively correlated with yield in the drought treatment. Conversely, the index is unrelated to yield under well-watered treatment, indicating that breeding for stress tolerance would not necessarily have a negative effect on the yield under non-stressed conditions. Based on the DTI value and yield under stress, the following clones were classified as drought tolerant and high yielding under stress: CIP396038.101, CIP396038.107, CIP396029.250, Gorebella, and CIP395112.32. Among these, CIP395112.32, CIP396038.107, and Gorebella were the best yielders both under drought and well-watered conditions. The genotype CIP396029.250 has the additional merit of being late blight resistant [36] and CIP396038.107, of being a good combiner for late blight resistance [56]. Consequently, clone CIP396029.250 may be suitable for release in drought prone areas. Clone CIP396038.107 could also be a good parent to generate progenies with combined drought tolerance and late blight resistance. The study showed that the three local cultivars—Gorebella, Belete, and Guassa—had moderate to high levels of drought tolerance. This could be associated with their adaptability to drought, since they were selected for yield stability across a broad range of environments with variability in rainfall amount and distribution [11, 57].

### Gene action and combining ability

The study showed highly significant GCA and SCA effects for total tuber yield, marketable tuber yield, average tuber weight, plant height, groundcover, and chlorophyll content, indicating the importance of both additive and non-additive gene action in conditioning these traits. Larger GCA than SCA effects for total tuber yield (60%), marketable tuber yield (68%), average tuber weight (68%), plant height (83%), chlorophyll content (83%), and groundcover (60% of total variance), indicated that additive genetic effects predominantly control the phenotypic variation of these traits under drought stress. There have been few studies on the inheritance of potato yield, yield components, and drought related traits under moisture stress. A recent report showed the predominance of additive genetic variance over non-additive for average tuber weight [47]. In wheat, additive gene effects have been demonstrated in the genetic control of flag leaf area duration, which corresponded to the ‘stay green’ effect [58]. Three quantitative trait loci (QTL) for chlorophyll content have been identified in potato [54]. These traits can be effectively improved by appropriate selection procedures.

The study showed that the following five parental clones: CIP396038.107, CIP396034.103, CIP396012.288, CIP395109.34, and CIP395112.32 exhibited a high GCA for yield and the most desirable traits for drought tolerance, and were parents of the most resistant families as measured by DTI. The yield potential of clones CIP395112.32, and CIP396034.103 was confirmed by their high *per se* performance under well-watered conditions. On the other hand, parental clone CIP395109.34, which showed undesirable GCA for chlorophyll content, was among the lowest yielding genotypes under well-watered conditions, suggesting that it could be associated with undesired “static” yield stability which results in reduced yield when high rainfall occurs. Cultivars with minimal yield losses under drought might have a low yield potential if their resistance is associated with stay green [22]. Nevertheless, these cultivars might harbour interesting drought tolerance traits that could be transferred to higher yielding commercial varieties. In addition, clones CIP395017.229, CIP395109.7, and CIP396031.108, which had significant and negative GCA for chlorophyll content, can also be utilized in breeding for high yields, because the loss of chlorophyll content was found to be correlated with yield both under stress and well-watered condition.

Families from crosses CIP395109.34 x CIP396041.102, CIP395096.2 x CIP396012.288, CIP395109.7 x CIP395017.14, and CIP396031.108 x CIP395017.14 were among the most drought tolerant and showed significant SCA effects for most yield and drought related traits. Most of the crosses with high yield and drought tolerance were obtained from parental combinations with different desirable characters. For instance, the high SCA observed in crosses involving CIP395096.2 and CIP395017.14 could probably be explained by the parents’ complementary effects for high root dry mass (measured in separate experiment as explained in S1 and S2 Tables) with the desired GCA of their counterparts for yield components and chlorophyll content, respectively. Larger and deeper roots provide better access to the remaining soil water. Different combinations of drought tolerance traits may lead to the same effect, namely tuber yield maintenance under drought conditions [22]. Effective crop improvement for drought tolerance will require the pyramiding of many complementary characters, with different combinations [2]. Among the best yielders and drought tolerant families, CIP395096.2 x CIP396012.288 showed a good SCA effect for late blight resistance [56], suggesting that some offspring from this combination would be high yielding, drought tolerant, and late blight resistant.

## Conclusions

The present study showed that clones CIP396034.103, CIP396012.288, and CIP395112.32 were good combiners for tuber yield under stress and most drought related traits. Families from crosses of CIP395109.34 x CIP396041.102, CIP395096.2 x CIP396012.288, CIP395109.7 x CIP395017.14, and CIP396031.108 x CIP395017.14 exhibited the best SCA effects for high yield and drought tolerance. The selected parents and families are good candidates to develop improved potato varieties for drought prone areas of north-western Ethiopia or similar environments. The predominance of variance due to GCA over SCA suggests that a high response to selection could be obtained either by directly selecting for yield or for desirable traits correlated with yield. The traits that showed strong correlation with yield under water stress were marketable tuber yield, marketable tuber number, average tuber weight, groundcover, and chlorophyll content. Overall, the results indicated that it will be possible to breed improved potato cultivars that combined drought tolerance, high yield potential, and resistance to late blight.

## Supporting information

S1 Table Analysis of variance of 25 potato clones for root tuber mass planted at Injibara, Ethiopia during 2013/2014 dry season under terminal water stress condition. (DOCX)

S2 Table Mean root dry mass of 25 potato clones evaluated at Injibara during 2014/2015 dry season under terminal water stress. (DOCX)

S3 Table Association among phenological and tuber yield. (DOCX)

S4 Table Multiple regression of yield under stress on yield under non-stress and the number of days from planting to 50% of plants exhibiting flower bud formation under well-watered treatments. (DOCX)

S5 Table Analysis of variance and mean values of traits in 32 families under drought conditions. (DOCX)

S6 Table Genetic distance estimates of 18 potato genotypes revealed by 23 SSR markers. (DOCX)

S1 Fig Chlorophyll content measured in 30 individual plants of 32 families and two replications under water stressed condition. (TIF)

S2 Fig Chlorophyll content measured in 60 individuals of 32 families under water stressed condition. (TIF)

S1 Dataset Raw data. (XLSX)
