# Supplementary material for: Combining ability of highland tropic adapted potato for tuber yield and yield components under drought
Source: PLoS One. 2017 Jul 25;12(7):e0181541. doi: 10.1371/journal.pone.0181541 (PMC5526565; doi:10.1371/journal.pone.0181541)
Supplement: S1 Table — (DOCX) [file pone.0181541.s001.docx]

**S1 Table. Analysis of variance of 25 potato clones for root dry mass^a^ planted at Injibara, Ethiopia during 2013/2014 dry season under terminal water stress condition**

| **Source of variation** | **d.f. (m.v.)** | **mean squares** |
| --- | --- | --- |
| Replication | 2 | 299.26 |
| Genotype | 24 | 91.4*** |
| Residual | 14(22) |  |
| **CV (%)** | **28.6** |  |

d.f, degrees of freedom; m.v, missing value.

^a^Root dry mass is from a previous field evaluation at Injibara, Ethiopia (10°57′ N, 36°56′ E, 2568 m). Twenty five clones evaluated during the dry season (November 11, 2013 to March 21, 2014) using 5 x 5 square lattice design with three replication of four rows, 3 m long plots, each having 40 plants, the inter- and intra-row spacing was 0.75 and 0.3m, respectively. Terminal drought stress was induced approximately at tuber initiation stage (45 days after planting). Roots of five plants (excluding tubers) were taken and dried in an oven (60 °C) to determine root dry mass of tested clones.
