# Supplementary material for: Combining ability of highland tropic adapted potato for tuber yield and yield components under drought
Source: PLoS One. 2017 Jul 25;12(7):e0181541. doi: 10.1371/journal.pone.0181541 (PMC5526565; doi:10.1371/journal.pone.0181541)
Supplement: S2 Table — (DOCX) [file pone.0181541.s002.docx]

**S2 Table. Mean root dry mass of 25 potato clones evaluated at Injibara during 2014/2015 dry season under terminal water stress**

| **clones** | **Root dry mass** |
| --- | --- |
| Shenkola | 5.1^a^ |
| CIP396034.103 | 9.1^a,b^ |
| Belete | 9.3^a-c^ |
| Abalolarge | 11.1^a-d^ |
| CIP396031.108 | 17.3^a-e^ |
| CIP395109.34 | 17.4^a-e^ |
| CIP393220.54 | 18.2^a-e^ |
| Enat beguaro | 19.4^b-e^ |
| Jalene | 20.0^b-e^ |
| CIP395077.12 | 20.3^b-e^ |
| CIP392633.64 | 21.0^b-e^ |
| CIP396004.263 | 21.2^b-e^ |
| CIP396038.101 | 22.3^c-e^ |
| CIP395015.6 | 23.5^de^ |
| Guassa | 23.8^de^ |
| Gorobella | 24.6^e^ |
| Gudene | 25.0^e^ |
| CIP396038.107 | 25.9^e^ |
| CIP395017.229 | 26.6^e^ |
| CIP395112.32 | 26.8^e^ |
| CIP396029.25 | 28.0^ef^ |
| CIP395017.14 | 40.8^fg^ |
| CIP395096.2 | 40.9^g^ |
| CIP396038.105 | 42.5^g^ |
| CIP395011.2 | 43.8^g^ |
| **Mean** | **23.4** |
| **CV (%)** | **28.6** |
