# Supplementary material for: Combining ability of highland tropic adapted potato for tuber yield and yield components under drought
Source: PLoS One. 2017 Jul 25;12(7):e0181541. doi: 10.1371/journal.pone.0181541 (PMC5526565; doi:10.1371/journal.pone.0181541)
Supplement: S3 Table — (DOCX) [file pone.0181541.s003.docx]

**S3 Table. Association among phenology and tuber yield.**

| Parameters | 50% bud formation DAP | Bud initiation DAP | Maturity-90 DAP | Maturity-110 DAP | TTY well-watered |
| --- | --- | --- | --- | --- | --- |
| Bud initiation DAP | 0.690*** | 1 |  |  |  |
| Maturity-90 DAP | -0.564*** | -0.446*** | 1 |  |  |
| Maturity-110 DAP | -0.400** | -0.347** | 0.834*** | 1 |  |
| TTY well-watered | -0.454*** | -0.348** | 0.390** | 0.283* | 1 |
| TTY stressed | -0.568*** | -0.443*** | 0.407*** | 0.330** | 0.600*** |

DAP, days after planting; TTY, total tuber yield.

***, **,* = significant at *P* < 0.001, *P* < 0.01 and *P* < 0.05, respectively
