# Supplementary material for: Combining ability of highland tropic adapted potato for tuber yield and yield components under drought
Source: PLoS One. 2017 Jul 25;12(7):e0181541. doi: 10.1371/journal.pone.0181541 (PMC5526565; doi:10.1371/journal.pone.0181541)
Supplement: S4 Table — (DOCX) [file pone.0181541.s004.docx]

**S4 Table. Multiple regression of yield under stress on yield under non-stress and the number of days from planting to 50% of plants exhibiting flower bud formation under well-watered treatments.**

| **Source** | **d.f.** | **Mean square** |
| --- | --- | --- |
| **Regression** | 2 | 0.068445*** |
| **Residual** | 46 | 0.003258 |
| **Total** | 48 | 0.005974 |
| **R^2^** | 45.5 |  |
| **Standard error of the estimate** | 0.0571 |  |
|  |  |  |
| **Parameter** | **Estimate** | **Standard error** |
| Constant | 0.478*** | 0.131 |
| TTY well-watered | 0.2715** | 0.0795 |
| 50% bud formation | -0.00646** | 0.00207 |

***, ** = significant at *P* < 0.001 and *P* < 0.01, respectively
