# Supplementary material for: Combining ability of highland tropic adapted potato for tuber yield and yield components under drought
Source: PLoS One. 2017 Jul 25;12(7):e0181541. doi: 10.1371/journal.pone.0181541 (PMC5526565; doi:10.1371/journal.pone.0181541)
Supplement: S5 Table — (DOCX) [file pone.0181541.s005.docx]

**S5 Table. Analysis of variance and mean values of traits in 32 families under drought conditions.**

| **Source of variation** | **d.f** | **TTY** | **MTY** | **ATW** | **GC** | **PHT** | **CC** |
| --- | --- | --- | --- | --- | --- | --- | --- |
|  |  | **Mean squares** | | | | | |
| Replication | 1 | 0.0003^ns^ | 1.21^ns^ | 104.1*** | 64.95^ns^ | 173.14*** | 19.31** |
| Family | 31 | 0.0109*** | 8.11*** | 109.85*** | 3314.60*** | 105.16*** | 12.81*** |
| Error | 31 | 0.0017 | 0.0015 | 3.06 | 23.95 | 8.29 | 2.19 |
| **CV** |  | 11.46 | 12.17 | 7.1 | 10.77 | 6.08 | 2.98 |
| **Mean** |  | **0.36** | **0.32** | **24.67** | **45.44** | **47.36** | **49.66** |

TTY, total tuber yield; MTY, marketable tuber yield; ATW, average tuber weight; PHT, plant height; GC, groundcover; CC, chlorophyll content; CV(%), coefficient of variance.

***, **,*, ns = significant at *P* < 0.001, *P* < 0.01, *P* < 0.05 and non-significant at *P* value 0.05, respectively
