# Supplementary material for: Combining ability of highland tropic adapted potato for tuber yield and yield components under drought
Source: PLoS One. 2017 Jul 25;12(7):e0181541. doi: 10.1371/journal.pone.0181541 (PMC5526565; doi:10.1371/journal.pone.0181541)
Supplement: S6 Table — (DOCX) [file pone.0181541.s006.docx]

**S6 Table. Genetic distance estimates of 18 potato genotypes revealed by 23 SSR markers.**

| **Clones** | **1** | **2** | **3** | **4** | **5** | **6** | **7** | **8** | **9** | **10** | **11** | **12** | **13** | **14** | **15** | **16** | **17** | **18** |
| --- | --- | --- | --- | --- | --- | --- | --- | --- | --- | --- | --- | --- | --- | --- | --- | --- | --- | --- |
| **1** |  |  |  |  |  |  |  |  |  |  |  |  |  |  |  |  |  |  |
| **2** | 0.33 |  |  |  |  |  |  |  |  |  |  |  |  |  |  |  |  |  |
| **3** | 0.29 | 0.29 |  |  |  |  |  |  |  |  |  |  |  |  |  |  |  |  |
| **4** | 0.39 | 0.33 | 0.29 |  |  |  |  |  |  |  |  |  |  |  |  |  |  |  |
| **5** | 0.41 | 0.36 | 0.32 | 0.41 |  |  |  |  |  |  |  |  |  |  |  |  |  |  |
| **6** | 0.34 | 0.32 | 0.36 | 0.46 | 0.34 |  |  |  |  |  |  |  |  |  |  |  |  |  |
| **7** | 0.37 | 0.36 | 0.34 | 0.46 | 0.36 | **0.52** |  |  |  |  |  |  |  |  |  |  |  |  |
| **8** | 0.27 | 0.33 | 0.37 | 0.34 | 0.33 | 0.28 | 0.29 |  |  |  |  |  |  |  |  |  |  |  |
| **9** | 0.34 | **0.27** | 0.29 | 0.38 | 0.37 | 0.28 | 0.29 | 0.30 |  |  |  |  |  |  |  |  |  |  |
| **10** | 0.32 | 0.33 | 0.31 | 0.35 | 0.30 | 0.38 | 0.44 | 0.33 | 0.27 |  |  |  |  |  |  |  |  |  |
| **11** | 0.32 | 0.32 | 0.39 | 0.31 | 0.35 | 0.33 | 0.32 | 0.34 | 0.33 | 0.33 |  |  |  |  |  |  |  |  |
| **12** | 0.34 | 0.28 | 0.28 | 0.32 | 0.29 | 0.27 | 0.29 | 0.29 | 0.27 | 0.29 | 0.36 |  |  |  |  |  |  |  |
| **13** | 0.35 | 0.32 | 0.32 | 0.33 | 0.41 | 0.28 | 0.31 | 0.32 | 0.32 | 0.31 | 0.35 | 0.35 |  |  |  |  |  |  |
| **14** | 0.47 | 0.33 | 0.34 | 0.42 | 0.44 | 0.40 | 0.41 | 0.32 | 0.39 | 0.32 | 0.34 | 0.33 | 0.36 |  |  |  |  |  |
| **15** | 0.42 | 0.34 | 0.33 | 0.43 | 0.40 | 0.35 | 0.40 | 0.35 | 0.38 | 0.38 | 0.35 | 0.36 | 0.39 | 0.50 |  |  |  |  |
| **16** | 0.32 | 0.33 | 0.29 | 0.33 | 0.32 | 0.24 | 0.29 | 0.34 | 0.32 | 0.27 | **0.26** | 0.27 | 0.29 | 0.32 | 0.33 |  |  |  |
| **17** | 0.36 | 0.46 | 0.32 | 0.38 | 0.35 | 0.32 | 0.37 | 0.37 | 0.30 | 0.34 | 0.29 | 0.29 | 0.36 | 0.36 | 0.39 | 0.43 |  |  |
| **18** | 0.32 | 0.39 | 0.29 | 0.36 | 0.38 | 0.30 | 0.30 | 0.31 | 0.30 | 0.32 | 0.32 | 0.36 | 0.32 | 0.36 | 0.35 | 0.38 | 0.44 |  |

1 = CIP395015.6, 2 = CIP395096.2, 3 = CIP395109.34, 4 = CIP395112.32, 5 = CIP396004.263, 6 = CIP396031.108, 7 = CIP396034.103, 8 = CIP395011.2, 9 = CIP395017.14, 10 = CIP395017.229, 11 = CIP395077.12, 12 = CIP396038.107, 13 = CIP392633.64, 14 = CIP393220.54, 15 = CIP393371.58, 16 = CIP396029.250, 17 = CIP396038.101, 18 = CIP396038.105; G = genotypes.
