# Supplementary figures and images for: Combining ability of highland tropic adapted potato for tuber yield and yield components under drought
Source: PLoS One. 2017 Jul 25;12(7):e0181541. doi: 10.1371/journal.pone.0181541 (PMC5526565; doi:10.1371/journal.pone.0181541)

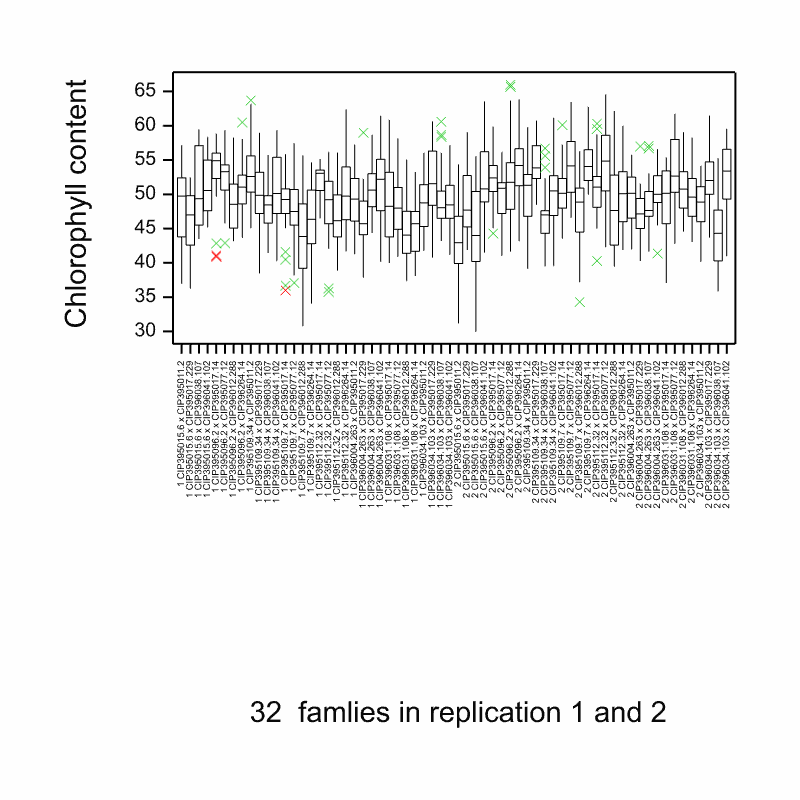

Supplement: S1 Fig — (TIF) [file pone.0181541.s007.tif]

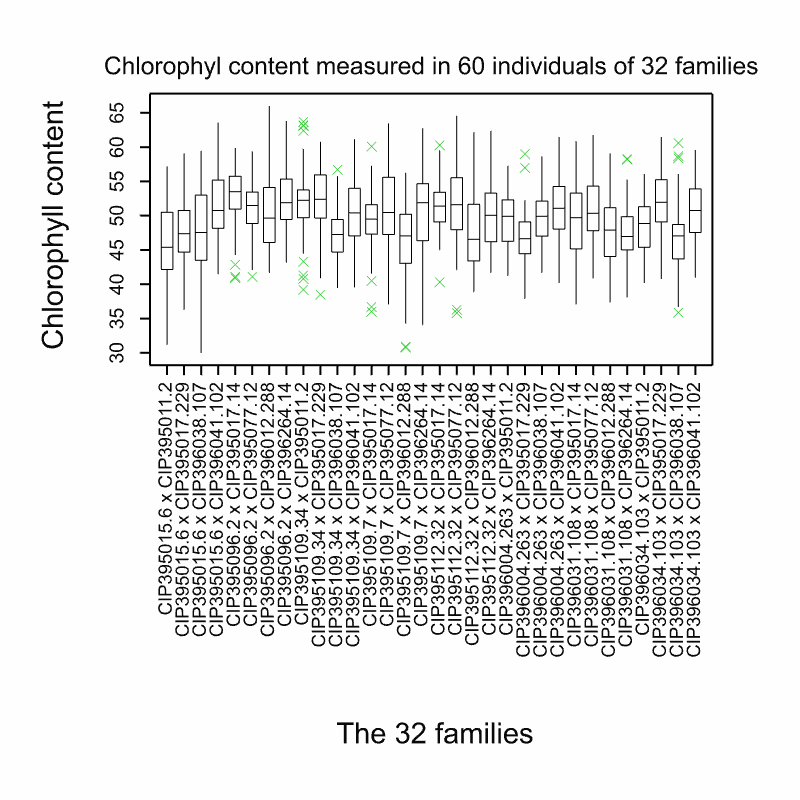

Supplement: S2 Fig — (TIF) [file pone.0181541.s008.tif]
